# The Influence of COVID-19 Pandemic on the Frequent Use of E-Cigarettes and Its Association with Substance Use and Mental Health Symptoms

**DOI:** 10.3390/bs12110453

**Published:** 2022-11-16

**Authors:** David Adzrago, Saanie Sulley, Lohuwa Mamudu, Cameron K. Ormiston, Faustine Williams

**Affiliations:** 1 Center for Health Promotion and Prevention Research, The University of Texas School of Public Health, The University of Texas Health Science Center at Houston, Houston, TX 77030, USA; 2 National Healthy Start Association, 1325 G Street, Washington, DC 20005, USA; 3 Department of Public Health, California State University, Fullerton, 800 N. State College Boulevard, Fullerton, CA 92831, USA; 4 Division of Intramural Research, National Institute on Minority Health and Health Disparities, Two White Flint North, Rockville, MD 20852, USA

**Keywords:** e-cigarette use, COVID-19 impact, substance use, mental health symptoms

## Abstract

Background: Although several studies examined the association between e-cigarettes, substance use, and mental health conditions, there is limited research on whether COVID-19-related stress and health outcomes, mental health symptoms, and substance use differ by the frequency of e-cigarette use during the COVID-19 pandemic. We assessed the association of past 30-day frequent use of e-cigarettes with alcohol, cannabis, anxiety/depression, and COVID-19 impact. Methods: We conducted a national online cross-sectional survey among a random sample of US adults aged 18 years or older (N = 5065) between 13 May 2021, and 9 January 2022. A multinomial logistic regression analysis was performed to assess the study aims. Results: Of the participants, 7.17% reported once to several times per month (OSTPM), 6.95% reported once to several times per week (OSTPW), and 6.57% reported every day to several times per day (ESTPD) use of e-cigarettes in the past month. Alcohol and cannabis use ESTPD and once to several times per week/month (OSTPW/M) were associated with a higher likelihood of e-cigarette use ESTPD and OSTPW/M, respectively. Anxiety/depression was associated with e-cigarette use ESTPD and OSTPW. Individuals who considered social distancing to be stressful were more likely to use e-cigarettes ESTPD and OSTPW/M compared to those that considered social distancing as not stressful. Conclusion: Individuals who engaged in the frequent use of alcohol or cannabis, had depression/anxiety, and considered social distancing to be stressful were more likely to engage in frequent e-cigarette use. Improving efforts geared toward reducing the use of substances may help decrease the health risks associated with e-cigarette use.

## 1. Introduction

Throughout the COVID-19 pandemic, United States (US) federal and state agencies implemented stay-at-home orders and restrictions on social gatherings [1]. Although essential, the sudden changes in social interaction, prolonged isolation while in quarantine, and other COVID-19-related stressors have likely contributed to the prevalence of depression and anxiety among the general US population [2,3,4]. Indeed, the American Psychological Association reported an increased demand for services related to depression and anxiety during the pandemic among US adults [5]. Reasons for increased mental health symptoms include job loss, food insecurity, disease anxiety, and housing instability [6]. In addition, 13–18% of Americans have initiated substance use or increased substance use to cope with stress during the pandemic [6,7]. Hispanics/Latinos, in particular, have reported a higher prevalence of substance use as a coping strategy compared to other racial/ethnic groups [6].

Among these substances are e-cigarettes. Studies both predating and during the pandemic indicate that individuals may use e-cigarettes or increase the frequency of e-cigarette use to relieve stress or cope with depression and anxiety symptoms [8,9,10]. E-cigarettes are electronic devices consisting of a battery, a cartridge filled with a fluid containing nicotine and other chemicals, and a heating element/atomizer that warms the liquid to create a vapor that can be inhaled through a mouthpiece [11]. In recent years, they have become increasingly popular among the general US population given they are advertised as less harmful to conventional cigarette smoking, are easier to obtain than cigarettes, available in many flavors, and used as a replacement for cigarettes [12,13]. They have even been recommended as options to aid in smoking cessation [14]. However, given the ease of access to e-cigarettes and potential for developing nicotine addiction, especially among the younger population, e-cigarettes may serve as a gateway to the development of tobacco use disorder (TUD) or other substance use disorders (SUDs) [15]. The risk factors for e-cigarette use include perceiving e-cigarettes to have lower relative risk than regular cigarettes and having anxiety, stress, family history of e-cigarette use, or peers who use e-cigarettes [9]. Impulsivity, post-traumatic stress disorder, and low self-esteem have also been linked with e-cigarette use [16]. Moreover, it has been noted that cigarette smoking, and alcohol and cannabis use often precede e-cigarette use [17]. Additionally, e-cigarette use is associated with cardiovascular complications and respiratory lung injury or disease [18,19,20]. In fact, the increase in hospitalizations due to e-cigarette product use-associated lung injury (EVALI) led to the declaration of EVALI as a public health emergency by the Centers for Disease Control and Prevention in 2019 [18].

Among the most vulnerable to SARS-CoV-2 infection are those who smoke or use e-cigarettes [21,22]. Some reports have found that the potential transmission of infection is associated with sharing and repetitive contact between smoking or vaping objects and proximity to the respiratory system [13,23]. A study by Gaiha et al. found that a positive COVID-19 diagnosis was associated with using e-cigarettes and the dual use of both e-cigarettes and cigarettes [24]. Moreover, studies have found that COVID-19 symptoms may be exacerbated or more frequent among individuals who use e-cigarettes [25,26].

Given the potential risk of developing SUDs among those who use e-cigarettes [27,28,29], the use of e-cigarettes to cope with stress, and the elevated prevalence of mental health symptoms during the COVID-19 pandemic, it is imperative to further our understanding on how the mental health of individuals who use e-cigarette has been affected during the pandemic. Although several studies have investigated the relationship between cigarettes, e-cigarettes, substance use, and mental health conditions [17,30,31], there is limited research on whether COVID-19-related stress, mental health symptoms, and substance use differ by the frequency of e-cigarette use during the COVID-19 pandemic. Our study aims to assess the association of the frequent use of e-cigarettes with alcohol, cannabis, anxiety/depression, and COVID-19 pandemic-related factors. Understanding the factors that contribute to the use of e-cigarettes and their relationship with substance use and mental health symptoms is essential to developing educational and treatment strategies that mitigate the mental and behavioral health effects of the pandemic.

## 2. Materials and Methods

### 2.1. Study Design and Samples

We used a national a 116-item online cross-sectional survey conducted among a random sample of US adults aged ≥18 years. The survey assessed substances (e.g., e-cigarette use, alcohol, cannabis), mental health (e.g., anxiety/depression), COVID-19 impact, and participants’ sociodemographic characteristics. Through the sponsorship of the National Institute of Health Division of Intramural Research Program, recruitment and survey distribution were performed by Qualtrics LLC between 13 May 2021 and 9 January 2022. There were 5413 samples with valid responses out of the 5938 participants that completed the survey. Our analytical samples for this study included 5065 participants with complete data on responses to the past 30-day frequent use of e-cigarette survey questions.

The National Institutes of Health—Intramural Research Program IRB—Human Research Protection Program—Office of Human Subjects Research Protections approved the research protocol on 23 December 2020, (IRB #000308) as exempt because it did not involve human subjects. A STROBE checklist for our study is available in Table S1.

### 2.2. Measures

The frequency of e-cigarette use during the last 30 days was the dependent variable in this study. This self-reported variable was measured by asking the participants how frequently they used e-cigarettes during the past month. The response options included “1 = not at all, 2 = once during the month, 3 = several times during the month, 4 = once a week, 5 = several times a week, 6 = every day or almost every day, or 7 = several times a day”. We recategorized the response options into four categories: “no use (i.e., option 1); once to several times per month [OSTPM] (i.e., options 2 and 3); once to several times per week [OSTPW] (i.e., options 4 and 5); and every day to several times per day [ESTPD] (i.e., options 6 and 7)”.

The self-reported independent variables included sociodemographic characteristics, the likelihood of contracting COVID-19, social interaction, substance use, and mental health symptoms. The sociodemographic characteristics were age (18–25, 26–34, 35–49, and 50+), gender identity (man, non-binary, transgender/else, and woman), sexual orientation (bisexual, else, gay, heterosexual, and lesbian), race/ethnicity (Asian, Black/African American, Latino/Hispanic, Other, and White), level of education completed (less than High School, High School diploma or GED, some college/vocational or technical school, and a college or higher degree), marital status (divorced/widowed/separated, married/living with a partner, and never been married), and annual household income (less than $25,000, $25,000 to <$35,000, $35,000 to <$50,000, $50,000 to <$75,000, and $75,000 or more).

The COVID-19 impact was assessed using a variety of questions. The likelihood of contracting COVID-19 was assessed by asking the participants, “Given your overall self-rated health, how likely do you feel that you will contract Coronavirus/COVID-19?” with response options of “not at all likely, slightly likely, moderately likely, very likely, and extremely likely.” We dichotomized this variable into “not likely and likely” (i.e., slightly likely, moderately likely, very likely, and extremely likely). The social interaction during and before the COVID-19 pandemic was measured with the question, “Compared to before the Coronavirus/COVID-19 pandemic, how often do you interact now with friends and relatives living outside your household?” The response options included “more than before, the same as before, and less than before.” The social distancing stress variable was determined with the question, “How stressful has social distancing been for you?” and the response options as “very stressful, somewhat stressful, a little stressful, and not at all stressful”. These were recategorized as “stressful” (i.e., very stressful, somewhat stressful, a little stressful, and not at all stressful).

The perceived general mental health status was determined using the question, “In general, how would you rate your overall mental health?” The response options were “poor, fair, good, very good, and excellent”. We dichotomized these response options into fair/poor and excellent/very good/good. Anxiety/depression were derived from the combination of the Generalized Anxiety Disorder 2-item (GAD-2) and Patient Health Questionnaire-2 (PHQ-2), which forms the PHQ-4 with a total score ranging from 0–12 [32,33]. The total score of ≥3 on the PHQ-4 scale denoted anxiety/depression and was used in this paper.

The past-month alcohol use frequency was measured using the question, “During the past month, have you had a drink containing alcohol?” The response options were “1 = “not at all; 2 = once during the month; 3 = several times during the month; 4 = once a week; 5 = several times a week; 6 = every day or almost every day; 7 = several times a day.” We recategorized the responses into four: “no use (i.e., option 1); OSTPM (i.e., options 2 and 3); OSTPW (i.e., options 4 and 5); and ESTPD (i.e., options 6 and 7). The past-month cannabis use frequency was also determined with the question, “During the past month, have you used marijuana?” and was similarly recategorized as that of the past month alcohol use frequency variable as described above.

## 3. Statistical Analysis

We first performed descriptive and bivariate analyses to estimate the prevalence and statistical differences in the past month of the frequent use of e-cigarettes by sociodemographic characteristics, the likelihood of contracting COVID-19, social interaction, social distancing stress, mental health symptoms, and substance use. The frequencies and percentages were estimated and reported for the descriptive statistics. The Chi-square test with a significance level of *p* < 0.05 was used to assess the statistical difference of the past month’s frequency of use of e-cigarettes between groups. Additionally, we used multinomial logistic regression analysis to model the past month’s frequency of use of e-cigarettes and assessed its association with the stated independent variables. We reported the relative risk ratio (RRR), 95% confidence intervals (CIs) as 2-tailed, and statistical significance of the independent variables based on *p* < 0.05. We used STATA/SE 16.1 to perform all the statistical analyses.

## 4. Results

### 4.1. Prevalence and Statistical Difference of E-Cigarette Use between Variables

Of the 5065 participants, 7.17% reported OSTPM, 6.95% reported OSTPW, and 6.57% reported ESTPD use of e-cigarette in the past month (see Table 1). Except for annual household income (*p* = ≥ 0.05), we observed statistically significant differences in the past month frequent use of e-cigarette across sociodemographic characteristics, COVID-19 pandemic impact, mental health symptoms, and substance use frequencies. Individuals aged 18–25 had the highest prevalence of e-cigarette use in the past month: OSTPM (9.71%), OSTPW (11.35%), and ESTPD (7.67%). For gender identity, those who identified as transgender/else had the highest prevalence in all the categories of e-cigarette use frequency. By sexual orientation, lesbian respondents reported the highest prevalence across frequency of use.

Individuals who self-identified as being likely to contract COVID-19 had higher prevalence rates of e-cigarette use across all frequency categories than those reporting themselves as not likely to contract COVID-19. The prevalence of e-cigarette use for all frequency categories was higher among those who indicated social distancing as stressful than those who indicated it as not stressful. Individuals experiencing more social interaction during the pandemic compared to before the pandemic had a higher prevalence of e-cigarette use across all frequency categories compared to individuals reporting less social interaction. Those who reported their general mental health as fair/poor had a higher prevalence of past month e-cigarette usage than those with excellent/very good/good across all usage frequencies and, similarly, among those who experienced anxiety/depression compared with those who did not experience anxiety/depression. Additionally, the prevalence of e-cigarette use was higher for all frequency rates among participants who reported alcohol use in the past month compared to respondents who did not report alcohol use. The trends were the same for cannabis use.

### 4.2. Assessing Association between the Independent Variables and the Frequent Use of E-Cigarette Sociodemographic Characteristics

The reference group for the past month of e-cigarette use was individuals that never used e-cigarettes (see Table 2). Compared to individuals aged 18–25 years, those aged 26–34 years (RRR = 0.60; 95% CI = 0.39–0.94) were less likely to use e-cigarettes OSTPM. Those aged 35–49 (RRRs = 0.23, 0.18, and 0.42) and ≥50 years (RRRs = 0.12, 0.11, and 0.21), compared to respondents aged 18–25 years, were less likely to use e-cigarettes OSTPM, OSTPW, and every ESTPD, respectively. Women, compared to men, were less likely to use e-cigarettes OSTPM (RRR = 0.54; 95% CI = 0.37–0.81) and ESTPD (RRR = 0.67; 95% CI = 0.45–0.98). Compared to heterosexual individuals, lesbian individuals were more likely to use e-cigarettes once to OSTPM (RRR = 3.71, 95% CI = 1.28, 10.75) and ESTPD (RRR = 4.00; 95% CI = 1.47–10.91). Black/African American (RRR = 0.39; 95% CI = 0.24–0.66), Asian (RRR = 0.28; 95% CI = 0.11–0.72), and Latino/Hispanic individuals (RRR = 0.37; 95% CI = 0.20–0.70) were less likely to use e-cigarettes ESTPD compared to White individuals. Participants who had college or higher degrees were less likely to use e-cigarettes OSTPM (RRR = 0.44; 95% CI = 0.20–0.97) than those who had less than High School education. The divorced/widowed/separated individuals were more likely to use e-cigarettes OSTPW (RRR = 2.42; 95% CI = 1.28–4.57) compared to those that had never been married.

### 4.3. COVID-19 Pandemic Impact

Those who reported their social interaction during the COVID-19 pandemic to be the same as before (RRR = 0.52; 95% CI = 0.31–0.89) or less than before (RRR = 0.45; 95% CI = 0.27–0.75), compared to more than before, were less likely to use e-cigarettes ESTPD. A lower likelihood of e-cigarette use OSTPM was also reported for those who considered their social interaction during the COVID-19 pandemic to be less than before (RRR = 0.54; 95% CI = 0.31–0.92). Those who regarded social distancing to be stressful were more likely to use e-cigarettes OSTPM (RRR = 2.30; 95% CI = 1.40–3.79), OSTPW (RRR = 1.78; 95% CI = 1.11–2.85), and ESTPD (RRR = 1.99; 95% CI = 1.28–3.09) compared to those that considered social distancing as not stressful.

### 4.4. Mental Health Factors

Anxiety/depression was associated with a higher likelihood of e-cigarette use OSTPW (RRR = 1.62; 95% CI = 1.06–2.49) and ESTPD (RRR = 1.69; 95% CI = 1.12–2.54).

### 4.5. Substance Use Frequency

Participants who reported alcohol use OSTPM had a higher likelihood of e-cigarette use OSTPM (RRR = 2.64; 95% CI = 1.64–4.24) and OSTPW (RRR = 3.90; 95% CI = 2.02–7.50). Higher likelihoods of e-cigarette use OSTPW and ESTPD were also observed for past month alcohol use OSTPW (RRR = 9.26, 95% CI = 4.88–17.60; RRR = 2.73, 95% CI = 1.73–4.31) and ESTPD (RRR = 3.29, 95% CI = 1.33–8.18; RRR = 2.79, 95% CI = 1.52–5.14). Respondents who use cannabis OSTPM (RRR = 7.40, CI = 4.63–11.81; RRR = 6.25, CI = 3.18–10.25; RRR = 2.43, CI = 1.36–4.33), OSTPW (RRR = 9.19, CI = 5.06–16.69; RRR = 19.31, CI = 11.47–32.51; RRR = 5.01, CI = 2.68–9.38), and ESTPD (RRR = 2.51, CI = 1.34–4.71; RRR = 2.89, CI = 1.55–5.40; RRR = 6.03, CI = 3.82–9.49) were associated with a higher likelihood of e-cigarette use OSTPM, OSTPW, and ESTPD, respectively.

## 5. Discussion

To the best of our knowledge, this study is among the first to assesses the association between the frequency of e-cigarette use and its association with other substance use, COVID-19-related factors, and mental health during the pandemic. Our study shows that individuals aged 18–25 had a higher frequency of e-cigarette use than people aged 26 or more, which mirrors the findings of other studies [34,35]. Additionally, lesbian individuals were four times more likely to have daily use of e-cigarette than heterosexual respondents, which is consistent with other studies that have found an increased likelihood of e-cigarette use among sexual minority communities [36,37,38]. The higher use of e-cigarettes among sexual minority persons such as lesbian individuals may be due to using e-cigarettes to cope with stressors such as discrimination, stigmatization, abuse, harassment, and health and social inequities [39,40,41,42]. This may be compounded by implicit cisnormative and heteronormative biases in health services and a lack of LGBTQ-specific interventions [40,41,43].

Divorced, widowed, or separated individuals were found to be more likely than married individuals to use e-cigarettes several times weekly. This finding is consistent with other studies that assessed the relationship between e-cigarettes and other tobacco-based products [44,45]. Previous literature indicates that divorced, widowed, and separated individuals report lower levels of social support and a higher frequency of loneliness, isolation, and psychological distress, which can predispose them to increased substance use, including e-cigarette use [46,47,48,49,50,51].

The overall prevalence of e-cigarette use (20.69%) in our study during the COVID-19 pandemic was higher than the prevalence (from 7.4% to 8.1%) reported in earlier studies before the pandemic [52], indicating e-cigarette use may have increased during the pandemic. False claims—many driven by the tobacco and nicotine industry—suggesting vaping provided protection against COVID-19 may have played a role in our study’s higher prevalence of e-cigarette use during the pandemic versus before the pandemic [53]. It is also plausible that COVID-19 stressors such as job loss, social isolation, cessation of normal life activities, and psychological distress may have resulted in the increased use of e-cigarettes as a coping strategy [54,55,56].

Individuals with less social interaction during the pandemic were less likely to use e-cigarettes than those with more interaction during the pandemic. This may be due to e-cigarette use being popular in social settings [57] and COVID-19 lockdowns preventing social groupings/gatherings, thereby hindering e-cigarette access. Thus, opportunities for e-cigarette use may have decreased [55,56,57,58,59]. Additionally, study participants who found social distancing stressful, used cannabis at any frequency, reported anxiety/depression, and used alcohol daily were more likely to report frequent e-cigarette use compared to their counterparts. These findings are consistent with previous studies that have established an association between e-cigarette use and SUDs and anxiety [7,27,29]. Given most studies on these associations utilized samples that comprised young adults or adolescents, we add to the existing literature by showing these trends among adults. The associations between social distancing stress and e-cigarette use may be linked to e-cigarettes being used to cope with this stress [60,61,62]. Similarly, respondents with depression/anxiety may have increased e-cigarette use as a form of self-medication [63].

Furthermore, our study highlights a relationship between alcohol consumption, cannabis, and e-cigarette use. There are numerous health risks associated with the co-use of alcohol and e-cigarettes and with the co-use of cannabis and e-cigarettes, including immune system damage and an increased risk of respiratory illnesses [64,65,66]. Co-use may therefore place individuals at greater risk for COVID-19 and subsequent adverse cardiovascular, pulmonary, and respiratory outcomes [67,68,69,70,71]. In fact, the co-use of e-cigarettes and cannabis has been shown to be associated with a positive COVID-19 diagnosis and COVID-19 symptoms [64]. Furthermore, the pandemic itself has shown to increase psychological distress and contracting COVID-19 can also lead to neuropsychiatric symptom manifestation (e.g., depression, anxiety, insomnia, post-traumatic stress disorder) [69]. The concurrent use of e-cigarettes and alcohol and/or cannabis may therefore lead to downstream adverse mental health outcomes, which can in turn increase substance use as a form of self-medication—creating a cycle of substance use and mental health symptoms [63]. Improving interventions or efforts geared toward reducing the use of these substances may help decrease the associated health risks, including the risks for COVID-19 and its immediate and long-term effects.

### Limitations 

This study cannot infer causality given the cross-sectional nature of the data. Detailed or granular information regarding the type or brand of e-cigarettes being used, the duration of use, and the mental health diagnosis among survey respondents were also not captured. The surveys were also conducted online and in English, meaning individuals without reliable access to the Internet or a computer and individuals with limited English proficiency may not be captured. Ultimately, however, our findings may aid in further understanding the dynamics between e-cigarettes, substance use, and mental health during the pandemic. 

## 6. Conclusions

The results of our study suggest an association between the frequency of e-cigarette use and social distancing stress, social interaction during the pandemic, substance use, and depression/anxiety during the pandemic. These findings can play a vital role in developing multistage educational approaches to increase knowledge and awareness and mitigate the potential harms of e-cigarette use and other substance use. Our study also highlights those with depression/anxiety, adults aged 18–25, and sexual minorities are at a higher risk for e-cigarette use. Future studies should also examine the link between e-cigarette use and other substances and mental health conditions.

## Figures and Tables

**Table 1:** Descriptive and bivariate analyses of the past-month e-cigarette use characteristics by sociodemographic characteristics, the likelihood of contracting COVID-19, social interaction, mental health symptoms, and substance use (N = 5065).

	Overall Sample	No Use	Once to Several Times per Month [OSTPM]	Once to Several Times per Week [OSTPW]	Every Day to Several Times per Day [ESTPD]	
	N (%)	n (%)	n (%)	n (%)	n (%)	*p*-Value
**Overall**		4017 (79.31)	363 (7.17)	352 (6.95)	333 (6.57)	
**Age Groups**						<0.001
18–25	978 (30.95)	697 (71.27)	95 (9.71)	111 (11.35)	75 (7.67)	
26–34	699 (22.12)	555 (79.40)	39 (5.58)	52 (7.44)	53 (7.58)	
35–49	1140 (36.08)	1053 (92.37)	24 (2.11)	22 (1.93)	41 (3.60)	
≥50	343 (10.85)	332 (96.79)	3 (0.87)	3 (0.87)	5 (1.46)	
**Gender Identity**						<0.001
Man	1783 (35.24)	1293 (72.52)	160 (8.97)	176 (9.87)	154 (8.64)	
Non-Binary	49 (0.97)	37 (75.51)	4 (8.16)	3 (6.12)	5 (10.20)	
Transgender/Else	42 (0.83)	26 (61.90)	5 (11.90)	5 (11.90)	6 (14.29)	
Woman	3186 (62.96)	2657 (83.40)	194 (6.09)	167 (5.24)	168 (5.27)	
**Sexual Orientation**						0.020
Bisexual	297 (5.90)	217 (73.06)	32 (10.77)	24 (8.08)	24 (8.08)	
Else	64 (1.27)	53 (82.81)	4 (6.25)	2 (3.12)	5 (7.81)	
Gay	89 (1.77)	66 (74.16)	5 (5.62)	9 (10.11)	9 (10.11)	
Heterosexual	4492 (89.18)	3594 (80.01)	308 (6.86)	305 (6.79)	285 (6.34)	
Lesbian	95 (1.89)	67 (70.53)	14 (14.74)	6 (6.32)	8 (8.42)	
**Race/Ethnicity**						<0.001
Asian	537 (10.60)	471 (87.71)	32 (5.96)	22 (4.10)	12 (2.23)	
Black/African American	1279 (25.25)	974 (76.15)	113 (8.84)	113 (8.84)	79 (6.18)	
Latino/Hispanic	948 (18.72)	748 (78.90)	84 (8.86)	67 (7.07)	49 (5.17)	
Other	207 (4.09)	150 (72.46)	20 (9.66)	20 (9.66)	17 (8.21)	
White	2094 (41.34)	1674 (79.94)	114 (5.44)	130 (6.21)	176 (8.40)	
**Level of Education Completed**						<0.001
Less than High School	290 (5.73)	200 (68.97)	42 (14.48)	25 (8.62)	23 (7.93)	
High School Diploma/GED	1186 (23.45)	925 (77.99)	90 (7.59)	74 (6.24)	97 (8.18)	
Some College/Vocational/Technical School	1664 (32.90)	1360 (81.73)	110 (6.61)	86 (5.17)	108 (6.49)	
College/Higher Degree	1918 (37.92)	1529 (79.72)	119 (6.20)	165 (8.60)	105 (5.47)	
**Marital Status**						<0.001
Divorced/Widowed/Separated	782 (15.49)	658 (84.14)	41 (5.24)	41 (5.24)	42 (5.37)	
Married/Living with a Partner	2706 (53.61)	2103 (77.72)	194 (7.17)	223 (8.24)	186 (6.87)	
Never been married	1560 (30.90)	1242 (79.62)	127 (8.14)	87 (5.58)	104 (6.67)	
**Annual Household Income**						0.194
<$25,000	1236 (24.64)	995 (80.50)	97 (7.85)	62 (5.02)	82 (6.63)	
From $25,000 to <$35,000	786 (15.67)	625 (79.52)	55 (7.00)	57 (7.25)	49 (6.23)	
From $35,000 to <$50,000	784 (15.63)	630 (80.36)	53 (6.76)	53 (6.76)	48 (6.12)	
From $50,000 to <$75,000	943 (18.80)	739 (78.37)	65 (6.89)	66 (7.00)	73 (7.74)	
≥$75,000	1268 (25.27)	998 (78.71)	89 (7.02)	107 (8.44)	74 (5.84)	
**Likelihood of Contracting COVID-19 Status**						0.002
Not at all likely	1605 (31.74)	1316 (81.99)	86 (5.36)	107 (6.67)	96 (5.98)	
Likely	3451 (68.26)	2694 (78.06)	276 (8.00)	244 (7.07)	237 (6.87)	
**Social Interaction During Compared to Before the COVID-19 Pandemic**						<0.001
More than before	632 (12.49)	387 (61.23)	92 (14.56)	77 (12.18)	76 (12.03)	
Same as before	2186 (43.20)	1748 (79.96)	158 (7.23)	154 (7.04)	126 (5.76)	
Less than before	2242 (44.31)	1878 (83.76)	112 (5.00)	121 (5.40)	131 (5.84)	
**Social Distancing Stress**						<0.001
Not at all stressful	1582 (31.28)	1407 (88.94)	53 (3.35)	51 (3.22)	71 (4.49)	
Stressful	3476 (68.72)	2605 (74.94)	309 (8.89)	301 (8.66)	261 (7.51)	
**Perceived General Mental Health Status**						0.015
Excellent/Good	3763 (74.84)	3009 (79.96)	262 (6.96)	267 (7.10)	225 (5.98)	
Fair/Poor	1265 (25.16)	980 (77.47)	98 (7.75)	81 (6.40)	106 (8.38)	
**Anxiety/Depression**						<0.001
No	3383 (66.82)	2897 (85.63)	172 (5.08)	169 (5.00)	145 (4.29)	
Yes	1680 (33.18)	1118 (66.55)	191 (11.37)	183 (10.89)	188 (11.19)	
**Past-Month Alcohol Use Frequency**						<0.001
No use	2125 (42.14)	1962 (92.33)	64 (3.01)	33 (1.55)	66 (3.11)	
Once to several times per month	1550 (30.74)	1156 (74.58)	193 (12.45)	119 (7.68)	82 (5.29)	
Once to several times per week	1071 (21.24)	707 (66.01)	78 (7.28)	172 (16.06)	114 (10.64)	
Every day to several times per day	297 (5.89)	177 (59.60)	26 (8.75)	25 (8.42)	69 (23.23)	
**Past Month Cannabis use Frequency**						<0.001
No use	3764 (74.89)	3415 (90.73)	128 (3.40)	97 (2.58)	124 (3.29)	
Once to several times per month	460 (9.15)	216 (46.96)	122 (26.52)	82 (17.83)	40 (8.70)	
Once to several times per week	333 (6.63)	104 (31.23)	65 (19.52)	117 (35.14)	47 (14.11)	
Every day to several times per day	469 (9.33)	258 (55.01)	42 (8.96)	50 (10.66)	119 (25.37)	

Statistical significance at *p* < 0.05. All *p*-values are based on Chi-square tests for the categorical variables. Differences in frequencies in categories may be due to missing data.

**Table 2:** Multinomial logistic regression analysis of past-month e-cigarette use and its associations with sociodemographic characteristics, the likelihood of contracting COVID-19, social interaction, substance use, and mental health symptoms.

	Base/Reference Category: No Use					
	Once to Several Times per Month [OSTPM]		Once to Several Times per Week [OSTPW]		Every Day to Several Times per Day [ESTPD]	
	RRR	95% CI	RRR	95% CI	RRR	95% CI
**Age Groups**						
18–25	Ref	-	-	-	-	-
26–34	0.60 *	(0.39, 0.94)	0.70	(0.46, 1.08)	0.96	(0.63, 1.46)
35–49	0.23 ***	(0.14, 0.39)	0.18 ***	(0.11, 0.31)	0.42 ***	(0.27, 0.67)
≥50	0.12 **	(0.04, 0.41)	0.11 ***	(0.03, 0.37)	0.21 **	(0.07, 0.62)
**Gender Identity**						
Man	Ref	-	-	-	-	-
Non-Binary	0.60	(0.04, 8.88)	0.93	(0.07, 12.73)	1.04	(0.09, 11.87)
Transgender/Else	2.53	(0.41, 15.46)	4.66	(0.82, 26.49)	1.43	(0.13, 15.66)
Woman	0.54 **	(0.37, 0.81)	0.71	(0.48, 1.05)	0.67 *	(0.45, 0.98)
**Sexual Orientation**						
Bisexual	0.88	(0.36, 2.14)	0.79	(0.32, 1.96)	0.74	(0.30, 1.82)
Else	2.00	(0.35, 11.36)	0.79	(0.07, 8.76)	1.29	(0.21, 7.93)
Gay	0.66	(0.17, 2.53)	1.43	(0.49, 4.14)	1.48	(0.54, 4.04)
Heterosexual	Ref	-	-	-	-	-
Lesbian	3.71 *	(1.28, 10.75)	1.17	(0.25, 5.52)	4.00 **	(1.47, 10.91)
**Race/Ethnicity**						
Asian	0.73	(0.34, 1.58)	0.50	(0.21, 1.20)	0.28 **	(0.11, 0.72)
Black/African American	0.88	(0.54, 1.43)	1.10	(0.68, 1.78)	0.39 ***	(0.24, 0.66)
Latino/Hispanic	1.17	(0.71, 1.93)	1.32	(0.79, 2.20)	0.37 **	(0.20, 0.70)
Other	1.41	(0.59, 3.37)	1.37	(0.53, 3.53)	1.02	(0.47, 2.22)
White	Ref	-	-	-	-	-
**Level of Education Completed**						
Less than High School	Ref	-	-	-	-	-
High School Diploma/GED	0.59	(0.26, 1.30)	1.06	(0.39, 2.88)	2.02	(0.71, 5.76)
Some College/Vocational/Technical School	0.50	(0.23, 1.06)	1.10	(0.42, 2.85)	1.42	(0.50, 3.99)
College/Higher Degree	0.44 *	(0.20, 0.97)	1.43	(0.54, 3.79)	1.38	(0.48, 4.00)
**Marital Status**						
Divorced/Widowed/Separated	1.48	(0.81, 2.71)	2.42 **	(1.28, 4.57)	1.34	(0.74, 2.40)
Married/Living with a Partner	1.17	(0.70, 1.95)	1.60	(0.92, 2.79)	1.25	(0.75, 2.06)
Never been married	Ref	-	-	-	-	-
**Annual Household Income**						
<$25,000	Ref	-	-	-	-	-
From $25,000 to <$35,000	0.71	(0.36, 1.40)	1.44	(0.74, 2.80)	0.88	(0.49, 1.61)
From $35,000 to <$50,000	0.90	(0.48, 1.69)	0.84	(0.41, 1.72)	0.55	(0.29, 1.07)
From $50,000 to <$75,000	1.24	(0.68, 2.26)	1.10	(0.56, 2.15)	1.14	(0.65, 2.01)
≥$75,000	0.91	(0.48, 1.75)	1.15	(0.57, 2.32)	0.82	(0.44, 1.52)
**Likelihood of Contracting COVID-19 Status**						
Not at all likely	Ref	-	-	-	-	-
Likely	1.52	(0.96, 2.41)	0.81	(0.54, 1.23)	0.80	(0.54, 1.18)
**Social Interaction During Compared to Before the COVID-19 Pandemic**						
More than before	Ref	-	-	-	-	-
Same as before	0.64	(0.37, 1.11)	0.76	(0.43, 1.33)	0.52 *	(0.31, 0.89)
Less than before	0.54 *	(0.31, 0.92)	0.61	(0.36, 1.05)	0.45 **	(0.27, 0.75)
**Social Distancing Stress**						
Not at all stressful	Ref	-	-	-	-	-
Stressful	2.30 **	(1.40, 3.79)	1.78 *	(1.11, 2.85)	1.99 **	(1.28, 3.09)
**Perceived General Mental Health Status**						
Excellent/Good	Ref	-	-	-	-	-
Fair/Poor	0.73	(0.45, 1.18)	0.65	(0.39, 1.08)	0.95	(0.61, 1.48)
**Anxiety/Depression**						
No	Ref	-	-	-	-	-
Yes	1.36	(0.89, 2.07)	1.62 *	(1.06, 2.49)	1.69 *	(1.12, 2.54)
**Past-Month Alcohol Use Frequency**						
No use	Ref	-	-	-	-	-
Once to several times per month	2.64 ***	(1.64, 4.24)	3.90 ***	(2.02, 7.50)	0.98	(0.59, 1.65)
Once to several times per week	1.55	(0.88, 2.73)	9.26 ***	(4.88, 17.60)	2.73 ***	(1.73, 4.31)
Every day to several times per day	1.68	(0.73, 3.86)	3.29 *	(1.33, 8.18)	2.79 **	(1.52, 5.14)
**Past-Month Cannabis Use Frequency**						
No use	Ref	-	-	-	-	-
Once to several times per month	7.40 ***	(4.63, 11.81)	6.25 ***	(3.81, 10.25)	2.43 **	(1.36, 4.33)
Once to several times per week	9.19 ***	(5.06, 16.69)	19.31 ***	(11.47, 32.51)	5.01 ***	(2.68, 9.38)
Every day to several times per day	2.51 **	(1.34, 4.71)	2.89 **	(1.55, 5.40)	6.03 ***	(3.82, 9.49)

RRR = Relative risk ratio. 95% CI = 95% confidence interval. Statistical significance at * *p* < 0.05, ** *p* < 0.01, and *** *p* < 0.001. Ref = reference.

## Data Availability

The data are not publicly available because the survey was collected for use by the Immigrant Health and Health Disparities (IHD) Research Laboratory, Division of Intramural Research, and National Institute on Minority Health and Health Disparities at the National Institutes of Health. We just started analyzing the data and cannot make it publicly available.

## Supplementary Materials

The following supporting information can be downloaded at: https://www.mdpi.com/article/10.3390/bs12110453/s1, Table S1: The influence of COVID-19 pandemic on the frequent use of e-cigarettes and its association with substance use and mental health symptoms—STROBE Checklist.
