# Supplementary material for: The Influence of COVID-19 Pandemic on the Frequent Use of E-Cigarettes and Its Association with Substance Use and Mental Health Symptoms
Source: Behav Sci (Basel). 2022 Nov 16;12(11):453. doi: 10.3390/bs12110453 (PMC9687156; doi:10.3390/bs12110453)
Supplement: Supplementary file 1 [file behavsci-12-00453-s001.zip › behavsci-1922347-supplementary.pdf]

The influence of COVID-19 pandemic on the frequent use of e-cigarettes and its association with substance use and mental health symptoms – STROBE Checklist

		Reporting Item	Page Number
**Title and abstract**			
Title	#1a	Indicate the study's design with a commonly used term in the title or the abstract	1
Abstract	#1b	Provide in the abstract an informative and balanced summary of what was done and what was found	2
**Introduction**			
Background / rationale	#2	Explain the scientific background and rationale for the investigation being reported	3-4
Objectives	#3	State specific objectives, including any prespecified hypotheses	4
**Methods**			
Study design	#4	Present key elements of study design early in the paper	4-5
Setting	#5	Describe the setting, locations, and relevant dates, including periods of recruitment, exposure, follow-up, and data collection	4-5
Eligibility criteria	#6a	Give the eligibility criteria, and the sources and methods of selection of participants	4-5
	#7	Clearly define all outcomes, exposures, predictors, potential confounders, and effect modifiers. Give diagnostic criteria, if applicable	5-6
Data sources / measurement	#8	For each variable of interest give sources of data and details of methods of assessment (measurement). Describe comparability of assessment methods if there is more than one group. Give information separately for exposed and unexposed groups if applicable.	5-6
Bias	#9	Describe any efforts to address potential sources of bias	4-5
Study size	#10	Explain how the study size was arrived at	4-5
Quantitative variables	#11	Explain how quantitative variables were handled in the analyses. If applicable, describe which groupings were chosen, and why	5-6
Statistical methods	#12a	Describe all statistical methods, including those used to control for confounding	6
Statistical methods	#12b	Describe any methods used to examine subgroups and interactions	N/A – no interaction analysis was performed
Statistical methods	#12c	Explain how missing data were addressed	N/A - Complete case analysis was performed

Statistical methods	#12d	If applicable, describe analytical methods taking account of sampling strategy	6
Statistical methods	#12e	Describe any sensitivity analyses	n/a
**Results**			
Participants	#13a	Report numbers of individuals at each stage of study—e.g. numbers potentially eligible, examined for eligibility, confirmed eligible, included in the study, completing follow-up, and analyzed. Give information separately for exposed and unexposed groups if applicable.	4-5
Participants	#13b	Give reasons for non-participation at each stage	4-5
Participants	#13c	Consider use of a flow diagram	N/A
Descriptive data	#14a	Give characteristics of study participants (e.g., demographic, clinical, social) and information on exposures and potential confounders. Give information separately for exposed and unexposed groups if applicable.	7-10
Descriptive data	#14b	Indicate number of participants with missing data for each variable of interest	N/A – complete case analysis was performed
Outcome data	#15	Report numbers of outcome events or summary measures. Give information separately for exposed and unexposed groups if applicable.	11-13
Main results	#16a	Give unadjusted estimates and, if applicable, confounder-adjusted estimates and their precision (e.g., 95% confidence interval). Make clear which confounders were adjusted for and why they were included	11-14
Main results	#16b	Report category boundaries when continuous variables were categorized	N/A
Main results	#16c	If relevant, consider translating estimates of relative risk into absolute risk for a meaningful time period	11-14
Other analyses	#17	Report other analyses done—e.g., analyses of subgroups and interactions, and sensitivity analyses	N/A
**Discussion**			
Key results	#18	Summaries key results with reference to study objectives	14-15
Limitations	#19	Discuss limitations of the study, taking into account sources of potential bias or imprecision. Discuss both direction and magnitude of any potential bias.	16
Interpretation	#20	Give a cautious overall interpretation considering objectives, limitations, multiplicity of analyses, results from similar studies, and other relevant evidence.	16

Generalizability	#21	Discuss the generalizability (external validity) of the study results	14-16
**Other Information**			
Funding	#22	Give the source of funding and the role of the funders for the present study and, if applicable, for the original study on which the present article is based	16
